# N-cadherin-ERα-Src signal models mediate the synergistic potentiation of activation of PI3K/Akt signal pathway in injured dopaminergic neurons by GDNF and E2

**DOI:** 10.1186/1750-1326-7-S1-S35

**Published:** 2012-02-07

**Authors:** Zixiao Shi, Meng Wang, Ye Xiong, Yaping Liu, Xiaozhou Wang, Jing Liu, Li Li, Dianshuai Gao

**Affiliations:** 1 Department of Neurobiology & Anatomy, Xu Zhou Medical College, Xuzhou Huaihaixilu No.84 Xu Zhou Medical College, Jiangsu Province, China 221002

## Background

Accumulating evidence indicates that glial cell line-derived neurotrophic factor (GDNF) synergizes with 17β-estradiol (E2) could protect dopaminergic neurons. However, the mechanisms have not yet been elucidated. Based on the fact that either E2 or GDNF can activate the intracellular PI3K/Akt signal pathway, we hypothesize that the synergic protection of dopaminergic neurons exerted by E2 and GDNF is ascribed to enhancing the activation of the cellular PI3K/Akt signal pathway in a certain way.

## Method

We studied the potential mechanism under the synergistic protective effects of E2 and GDNF on dopaminergic neurons using the MN9D cell line. The MN9D cells were treated with 6-OHDA before incubating with either E2 or GDNF or both. Endogenous AKT phosphorylation and precise underlying mechanistic studies were revealed using co-immunoprecipitation(co-IP), western blot and immuno-fluorescent staining.

## Result

Compared with the sole administration of GDNF or E2, the co-administration of GDNF and E2 significantly increased the Akt phosphorylation in injured dopaminergic neurons. Incubation of GDNF and E2 promoted the interaction of estrogenic α-receptor (ERα) with the intracellular N-cadherin which potentially recruited ERα to the inner surface of cell membrane. The GDNF and E2 mediated AKT phosphorylation was potentially mediated through an Src-dependent signaling pathway as inhibition of Src using specific inhibitor totally abrogated this process.

## Conclusion

The above findings indicated the potential importance of AKT in GDNF and E2 mediated synergistic protective effects in 6-OHDA injured MN9D cells. E2 recruited ERα to the inner surface of the cell membrane which could at least partially participate in the downstream AKT phosphorylation. We also proposed a role of Src as a potential mediator during this process. This study further explored the underlying protective mechanism of GDNF and E2 and has important clinical relevance.

